# Deep Auto-Encoder and Deep Forest-Assisted Failure Prognosis for Dynamic Predictive Maintenance Scheduling

**DOI:** 10.3390/s21248373

**Published:** 2021-12-15

**Authors:** Hui Yu, Chuang Chen, Ningyun Lu, Cunsong Wang

**Affiliations:** 1 Integrated System Integration Department, No. 38 Research Institute of CETC, Hefei 230088, China; yuhuihustac@163.com; 2 College of Automation Engineering, Nanjing University of Aeronautics and Astronautics, Nanjing 211106, China; chenchuang@nuaa.edu.cn; 3 Institute of Intelligent Manufacturing, Nanjing Tech University, Nanjing 210009, China; wangcunsong@njtech.edu.cn

**Keywords:** failure prognosis, maintenance decision-making, deep auto-encoder, deep forest, maintenance cost

## Abstract

Prognostics and health management (PHM) with failure prognosis and maintenance decision-making as the core is an advanced technology to improve the safety, reliability, and operational economy of engineering systems. However, studies of failure prognosis and maintenance decision-making have been conducted separately over the past years. Key challenges remain open when the joint problem is considered. The aim of this paper is to develop an integrated strategy for dynamic predictive maintenance scheduling (DPMS) based on a deep auto-encoder and deep forest-assisted failure prognosis method. The proposed DPMS method involves a complete process from performing failure prognosis to making maintenance decisions. The first step is to extract representative features reflecting system degradation from raw sensor data by using a deep auto-encoder. Then, the features are fed into the deep forest to compute the failure probabilities in moving time horizons. Finally, an optimal maintenance-related decision is made through quickly evaluating the costs of different decisions with the failure probabilities. Verification was accomplished using NASA’s open datasets of aircraft engines, and the experimental results show that the proposed DPMS method outperforms several state-of-the-art methods, which can benefit precise maintenance decisions and reduce maintenance costs.

## 1. Introduction

The rapid development of industrial Internet of Things technology greatly promotes the complexity, integration, and intelligence of modern engineering systems [1,2,3,4,5]. It also raises significant challenges in the safety and reliability of systems’ operation. Due to the unavoidable degradation of various components caused by wearing, aging, fatigue, functional design defects, and complicated environmental factors, the failure probability of the whole system is high and the consequences may be intolerable [6,7,8,9,10]. Precise predictive maintenance is an urgent need in those systems, especially for applications such as nuclear power plants, missile weapons, and aerospace vehicles, which have extremely high reliability and safety requirements. Therefore, prolonging the effective service life and ensuring the reliability with less maintenance cost is of great significance in practice. The key is to realize failure prognosis-based maintenance decision-making, that is, to predict the failure probabilities and carry out just-in-time maintenance activities [11,12,13,14,15].

In recent decades, failure prognosis has received extensive attention, and many research results have been achieved. For instance, an integrated feature-based failure prognosis method was developed in [16], where the dynamics of various failures were detected using signal processing technology, and the adaptive Bayesian algorithm was used to forecast the remaining useful life (RUL) of the faulty bearings. In [17], a multi-stream deep recurrent neural network was developed to handle the features extracted from the vibration signals for failure prognosis. In [18], a Kalman filter and particle filter were combined to predict the failure of satellite reaction wheels. A new hybrid fault prognosis method for multi-functional spoiler systems was proposed in [19], where distributed neural networks were used to estimate the failure parameters, and the recursive Bayesian algorithm was employed to anticipate the system RUL with the estimated failure parameters. Also, some time series-based forecasting methods have been combined with statistically based classification techniques to forecast the failures in cyber-physical systems [20].

However, the above studies only focused on the accuracy of failure prognosis and took no consideration on how to use these valuable failure prognostic results for maintenance decisions. Indeed, there are obvious advantages to jointly considering the two aspects of failure prognosis and maintenance decision-making. For example, in practice, the failure prognosis and maintenance decision-making can be regarded as a whole process affecting the safe operation of the system, where the accuracy of failure prognostics directly affects the effectiveness of maintenance decision-making. Accordingly, their joint research can provide important technical support for the integration of the control, decision-making, and management of complex engineering systems. For such a purpose, this paper develops a deep auto-encoder and deep forest-assisted failure prognosis method for dynamic predictive maintenance scheduling (DPMS). The proposed DPMS method involves a complete process from performing failure prognosis to making maintenance decisions. Firstly, representative features that can reflect system degradation are extracted from raw data by using a deep auto-encoder. Secondly, the features are processed by a deep forest network to compute the system failure probabilities in moving time horizons. Finally, these failure probabilities are used to compute the costs of possible maintenance decisions, and the maintenance activities are scheduled accordingly based on two rules.

The main contributions of this paper are highlighted as follows:

- A unified and powerful deep auto-encoder and deep forest integrated algorithm is proposed to handle the raw condition monitoring data. It can automatically extract the representative features reflecting system degradation and construct the mapping between the features and discrete degradation states for failure prognosis.
- Two decision rules are designed to deal with the DPMS. With the prognostic failure probabilities, the maintenance and inventory decisions can be made through quickly evaluating the costs of different decisions.
- With NASA’s open datasets of aircraft engines, the proposed DPMS method outperforms several state-of-the-art methods, which can benefit precise maintenance decisions and reduce maintenance costs.

The remainder of this paper is structured as follows. In Section 2, the proposed deep auto-encoder and deep forest-assisted failure prognosis and maintenance decision-making method will be described in detail. Section 3 will validate the effectiveness of the proposed methodology, and its performance will be highlighted by comparing it with several state-of-the-art methods. The last section concludes this paper.

## 2. Methodology

### 2.1. Key Idea

**Figure 1:**
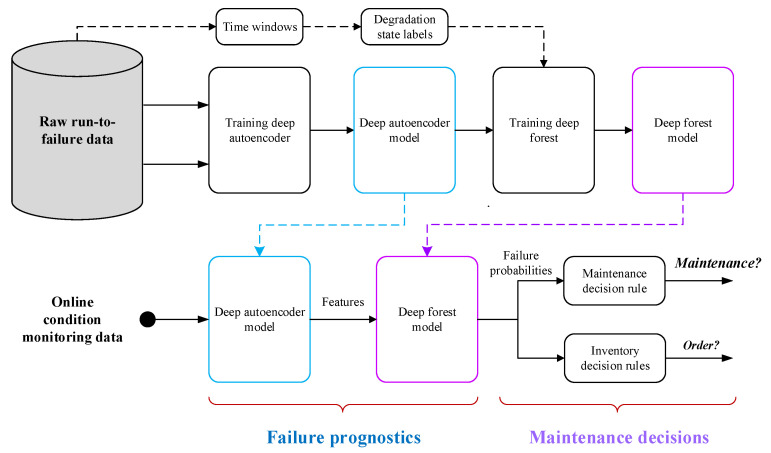
Proposed predictive maintenance framework.

The proposed methodology is based on real-time condition monitoring data (such as temperature, pressure, and rotational speed) collected by multiple sensors installed in the system. It contains two parts, failure prognosis and maintenance decision-making, and the complete process from performing failure prognosis to making maintenance decisions is shown in Figure 1.

In the failure prognosis stage, the use of a deep auto-encoder is proposed to extract the representative features reflecting system degradation from sensor data. Once the representative features are obtained, the mapping between features and degradation states can be constructed by using the deep forest algorithm, where the degradation states are determined according to the requirements of operational planners. As an illustration, if the operational planners require the failure information in two different time windows ($w_{1}$ and $w_{2}$), the degradation process of the system will be divided into three discrete states (Deg1, Deg2, and Deg3). Deg1 denotes the case where the system RUL is greater than $w_{2}$, i.e., $RUL>w_{2}$, whereas Deg3 represents the case where the system RUL is less than $w_{1}$, i.e., $RUL\leq w_{1}$. Deg2 refers to the case where the system RUL is in the period $\left(w_{1},w_{2}\right]$, i.e., $w_{1}<RUL\leq w_{2}$. Compared with Deg1 and Deg2, the degradation of Deg3 is more serious.

In the maintenance decision-making stage, the online condition monitoring data are first fed into the well-trained deep auto-encoder model to produce the representative features of the system degradation. With the representative features as the input, the well-trained deep forest model will output the failure probabilities belonging to different degradation states. Finally, the failure probabilities are used to compute the costs of different decisions, and the maintenance and inventory activities are scheduled according to two decision cost-based rules.

### 2.2. Degradation Feature Extraction Using a Deep Auto-Encoder

Auto-encoder technology is an important branch of deep learning theory and can be regarded as a feature extraction method of isodimensional mapping [21]. A basic auto-encoder consists of two main parts: an encoder and a decoder. The function of the encoder is to encode the high-dimensional input $x\in R^{D}$ into a low-dimensional implicit variable $h\in R^{d}$ (d<D), so as to force the neural network to learn the most informative features. The function of the decoder is to restore the hidden variable h of the code layer to the initial dimension. The best state is that the output $\tilde{x}\in R^{D}$ of the decoder can perfectly or approximately restore the original input $x\in R^{D}$, i.e., $\tilde{x}\approx x$. Therefore, the implicit variable $h\in R^{d}$ can be considered as the representative feature reflecting system degradation.

**Figure 2:**
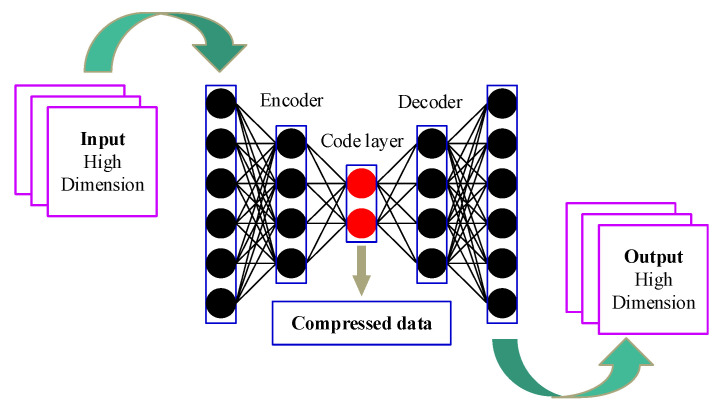
Deep auto-encoder architecture.

In this paper, a deep structure of a basic auto-encoder, known as a deep auto-encoder, was employed to extract deeper representative degradation features. As shown in Figure 2, the deep auto-encoder contains several hidden layers. For an L-layer deep auto-encoder, the encoding process of the original data x from the input layer to the l-th hidden layer can be expressed as follows:

(1) $$\{\begin{array}{l} h^{\left(0\right)}=x\\ h^{\left(l\right)}=f_{e}^{\left(l\right)}\left(W_{e}^{\left(l\right)}h_{e}^{\left(l-1\right)}+b_{e}^{\left(l\right)}\right) \end{array}$$

where $f_{e}^{\left(l\right)}:R^{D}\to R^{d}$ refers to the encoding function and $W_{e}^{\left(l\right)}$ and $b_{e}^{\left(l\right)}$ are the weight and bias of the encoding layer. The decoding process of the implicit variable $h^{\left(L\right)}$ from the code layer to the output layer is

(2) $$\{\begin{array}{l} {\tilde{x}}^{\left(L+1\right)}=h^{\left(L\right)}\\ {\tilde{x}}^{\left(l\right)}=f_{d}^{\left(l\right)}\left(W_{d}^{\left(l\right)}{\tilde{x}}^{\left(l+1\right)}+b_{d}^{\left(l\right)}\right) \end{array}$$

where $f_{d}^{\left(l\right)}:R^{d}\to R^{D}$ refers to the decoding function and $W_{d}^{\left(l\right)}$ and $b_{d}^{\left(l\right)}$ are the weight and bias of the decoding layer. Commonly, the sigmoid function can be used for the encoding function $f_{e}^{\left(l\right)}$ and decoding function $f_{d}^{\left(l\right)}$. It is given by

(3) $$sigmoid\left(x\right)=\frac{1}{1+e^{-x}}$$

To determine the deep auto-encoder parameters $\left\{W_{e}^{\left(l\right)},W_{d}^{\left(l\right)},b_{e}^{\left(l\right)},b_{d}^{\left(l\right)}\right\}$, a loss function is designed as follows:

(4) $$J\left(W,b\right)=\frac{1}{2M}\overset{M}{\underset{i=1}{\sum }}\|{\tilde{x}}_{i}-x_{i}{\|}_{2}^{2}+\frac{\lambda }{2}\overset{L-1}{\underset{l=1}{\sum }}\overset{s_{l+1}}{\underset{i=1}{\sum }}\overset{s_{l}}{\underset{j=1}{\sum }}{\left(W_{ij}^{\left(l\right)}\right)}^{2}$$

where M is the number of training samples, λ refers to the weight decay parameter, $s_{l}$ represents the number of nodes in layer l, and $W_{ij}^{\left(l\right)}$ is the weight coefficient of the propagation path of the j-th neuron in layer l and the i-th neuron in layer l+1. In Equation (4), the first term refers to the reconstruction error of the deep auto-encoder, while the second term is used to prevent over-fitting. Finally, Equation (4) can be minimized by using the stochastic gradient descent method.

### 2.3. Failure Prognosis Using Deep Forest

To capture the nonlinear relationship between the representative features and discrete degradation states, an integrated learning method called deep forest [22] is employed. One of the main advantages of the deep forest is the ability to handle unbalanced observation data. As mentioned in Section 2.1, the degradation process of a system can be divided into three discrete states (Deg1, Deg2, and Deg3) according to its RUL. Considering that the system operates under normal conditions most of the time, the amount of data of Deg1 will be significantly more than those of Deg2 and Deg3. Therefore, when applying the deep forest for system failure prognosis, it can improve the prognostic accuracy by handling the unbalanced observation data.

**Figure 3:**
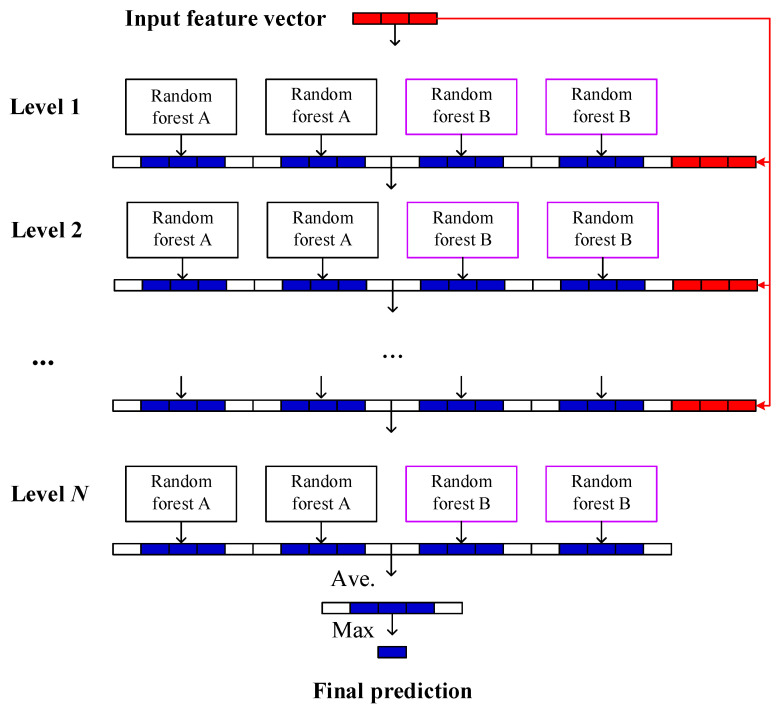
Structure diagram of deep forest prognosis model.

The schematic diagram of the deep forest prognosis model constructed in this paper is shown in Figure 3. Similar to the layer expansion of the deep neural network (DNN), the deep forest is formed by the automatic cascading expansion of a multi-layer forest structure (from level 1 to level *N*), without the need to set the number of forest layers in advance. Each layer of forest usually receives the feature vector generated from the previous layer, and then outputs the generated feature vector to the next layer. In the construction of each layer of the model, two kinds of random forests are contained, i.e., complete random forest (noted as random forest A) and ordinary random forest (noted as random forest B). The complete random forests consist of multiple decision trees, and each tree contains all the features. A feature is randomly selected as the split node of the split tree, and the splitting process is not terminated until each leaf node contains only one category or no more than 10 samples. The ordinary random forests randomly select $\sqrt{d}$ (d is the input feature dimension) candidate features and then employs the Gini index [23] to select split nodes for the growth of the tree. 

Given a sample set V, the Gini index can be calculated by

(5) $$Gini\left(V\right)=1-\overset{G}{\underset{g=1}{\sum }}{\left(\frac{V_{g}}{V}\right)}^{2}$$

where $V_{g}$ is a subset of samples belonging to the g category, and G is the number of categories. If the sample set V is divided into two parts, $V_{1}$ and $V_{2}$ ($V=V_{1}+V_{2}$), according to whether the feature A takes a certain possible value a, then under the condition of the feature A, the Gini index of the sample set V (denoted by Gini(V,A)) can be defined as

(6) $$Gini\left(V,A\right)=\frac{\left|V_{1}\right|}{\left|V\right|}Gini\left(V_{1}\right)+\frac{\left|V_{2}\right|}{\left|V\right|}Gini\left(V_{2}\right)$$

Gini(V,A) represents the uncertainty of the sample set V through the feature A=a segmentation. Similar to entropy, the greater the Gini(V,A) value, the greater the uncertainty. Thus, the feature value with the minimum Gini(V,A) will be chosen, i.e.,

(7) $$a^{*}=\arg_{a\in A}\min Gini\left(V,A=a\right)$$

**Figure 4:**
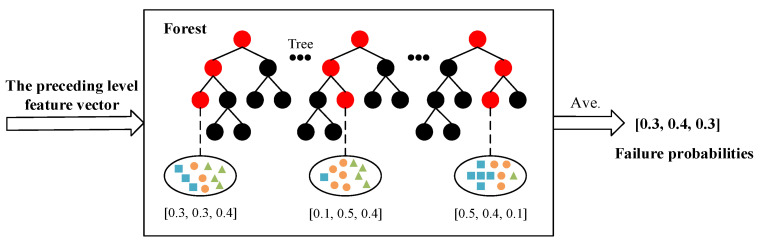
Structure diagram of class vector generation in deep forest.

According to the optimal segmentation feature value $a^{*}$, each decision tree can continuously divide subspaces in the feature space, and each subspace is labeled. Then, the leaf nodes can obtain the probabilities of different categories in the training sample, and the proportions of the various types in the entire forest will be produced by averaging the various proportions of all decision trees in each forest, as shown in Figure 4. In the training process of the cascade forest, the generated category distribution vector will be connected with the original input vector to feed the lower cascade layer through the cascade channel. When the classification accuracy of the verification set converges or reaches the expected value, the model training is terminated. It should be noted that the deep forest prognosis model constructed in this paper is different from many decision tree algorithms and does not require a pruning step. This is mainly because the deep forest is formed through the integration of random forests, and the random sampling and random feature selection of the random forest strengthen the diversity of the integrated learning and the generalization ability of the overall model. In other words, even if the deep forest is not pruned, there may be no over-fitting.

### 2.4. Maintenance-Related Decision Rules Based on Prognostic Information

Within the proposed predictive maintenance framework, the basic decisions that need to be made at each monitoring point are whether to place a spare part order or maintain the system. Actually, due to the technical and logistical constraints, it is difficult to perform maintenance on the spot in real time. In this paper, maintenance actions are considered perfect and decisions are only made at each inspection moment. Given the failure prognostic information ($P(RUL>w_{2})$, $P(w_{1}<RUL\leq w_{2})$, and $P\left(RUL\leq w_{1}\right)$), the following two maintenance-related decision rules [24] are designed:

- Inventory decision: The inventory decision aims to determine whether to place a spare part order at the current inspection time (*h*-th inspection period for example). For an option to order the spare part, the cost is computed by

(8) $$\begin{array}{c} C_{o}=C_{ins}\cdot \overset{+\infty }{\underset{i={\left[w_{2}/\Delta T\right]}^{+}}{\sum }}P\left(\left(i-1\right)\Delta T<RUL\leq i\Delta T\right)\cdot \left(i\Delta T-w_{2}\right)\\ \;\;\;\;\approx P(RUL>w_{2})\cdot C_{ins}{\left[\frac{\max({\overset{-}{T}}_{f}-h\Delta T-w_{2},\Delta T)}{\Delta T}\right]}^{+}\Delta T \end{array}$$

where $C_{ins}$ represents the inventory cost of the spare part per unit time, $w_{2}={\left[L/\Delta T\right]}^{+}\cdot \Delta T$ is the time window associated with the lead time L, ΔT is a fixed inspection interval, ${\overset{-}{T}}_{f}$ is the mean time of system failure, and ${\left[\cdot \right]}^{+}$ means taking an upper integer. On the contrary, the cost incurred by not ordering the spare part is given by

(9) $$C_{no}=C_{os}\cdot P(w_{2}<RUL\leq w_{2}+\Delta T)$$

where $C_{os}$ refers to the out-of-stock cost. Notably, the cost of not ordering a spare part is not the actual charged cost. It is considered as a damage estimation with wrong decision. With the computed $C_{o}$ and $C_{no}$, the inventory decision can be made by

(10) $$Order=\{\begin{array}{l} 0,\;\;C_{no}<C_{o}\\ 1,\;\;\;others \end{array}$$

where Order=0 means that there is no need to order a spare part at the current inspection time, whereas Order=1 requires a spare part order to be placed.

- Maintenance decision: The maintenance decision aims to determine whether to maintain the system at the current inspection time. For an option to maintain the system, the cost rate is computed by

(11) $$C_{m}=\frac{C_{p}+I\left(S_{h}=0\right)\cdot C_{os}}{h\Delta T}$$

where $C_{p}$ refers to the preventive cost, $S_{h}$ denotes the storage state, and I(⋅) is an indicator function, where I(⋅)=1 when the condition is met and I(⋅)=0 otherwise. On the contrary, the cost rate incurred by not maintaining the system is given by

(12) $$C_{nm}=\frac{P\left(RUL\leq w_{1}\right)\cdot \left(C_{c}+I\left(S_{h}=1\right)\cdot C_{ins}\Delta T+I\left(S_{h+1}=0\right)\cdot C_{os}\right)}{\left(h+1\right)\Delta T}$$

where $C_{c}$ refers to the corrective cost and $w_{1}=\Delta T$ is the time window associated with the inspection interval ΔT. With the computed $C_{m}$ and $C_{nm}$, the maintenance decision can be made by

(13) $$Maintenance=\{\begin{array}{l} 0,\;\;C_{nm}<C_{m}\\ 1,\;\;\;others \end{array}$$

where Maintenance=0 means that there is no need to perform maintenance activities at the current inspection time, whereas Maintenance=1 requires maintenance to the system.

### 2.5. Implementation Process of Predictive Maintenance

For an in-service system under consideration, the proposed DPMS method can be implemented by the following procedures:

- Obtain the real-time condition monitoring data from multiple sensors installed in the system;
- Obtain the representative features that can reflect system degradation using the deep auto-encoder;
- Produce the failure probabilities in moving time horizons using deep forest;
- Compute the costs of different decisions, and schedule maintenance and inventory activities according to two decision cost-based rules.

To evaluate the performance of the proposed DPMS method, the prognostic accuracy and maintenance cost rate reflecting the economic benefits of the system are adopted. The prognostic accuracy can be mathematically stated as

(14) $$Accuracy=\frac{1}{N}\overset{N}{\underset{i=1}{\sum }}C_{i}$$

where $C_{i}=1$ if ${\hat{y}}_{i}=y_{i}$ and $C_{i}=0$ otherwise, N is the number of test samples, ${\hat{y}}_{i}$ is the predicted category of the i-th sample, and $y_{i}$ is the true category. The maintenance cost rate is defined as the ratio of total maintenance cost to total running time [25], i.e.,

(15) $$MCR=\{\begin{array}{c} \frac{C_{p}+I\left(S_{h}=0\right)\cdot C_{os}}{h\cdot \Delta T},\;\mathrm{preventive}\;\mathrm{maintenance}\;\mathrm{is}\;\mathrm{performed}.\\ \frac{C_{c}+I\left(S_{h}=0\right)\cdot C_{os}}{h\cdot \Delta T},\;\mathrm{corrective}\;\mathrm{maintenance}\;\mathrm{is}\;\mathrm{performed}. \end{array}$$

The strategy with the lower maintenance cost rate is considered to have a better performance.

## 3. Results

The described DPMS method was implemented on Python 2.7 software, where the “gcForest” toolbox available at GitHub platform [26] was used. To validate its performance, the C-MAPSS dataset available in NASA’s data repository [27] was considered.

### 3.1. Description of the C-MAPSS Dataset

**Figure 5:**
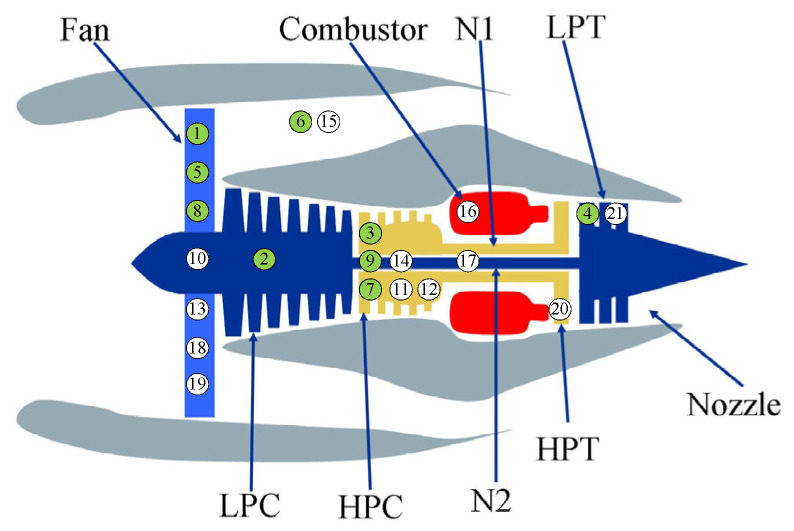
Structure diagram of the simulated engine [33].

The C-MAPSS dataset is a popular dataset simulating various scenarios of aircraft engine degradation and has been widely used to test the performance of various data-driven failure prognosis methods [28,29,30,31,32]. Figure 5 shows the schematic diagram of the simulated engine. It is composed of several main components, such as a fan, low-pressure compressor (LPC), combustor, high-pressure compressor (HPC), low pressure turbine (LPT), high pressure turbine (HPT), and nozzle. In the initial phase of each scenario, the engine operated normally. As the service time increased, its performance degraded gradually, and the complete run-to-failure data have been recorded in the “train-FD001.txt” subset. In this paper, to determine the structure of deep forest and test the decision accuracy of the engine at different inspection moments, the “train-FD001.txt” subset including 100 trajectories will be divided into three parts. The first part consisting of the first 70 trajectories is used to train the proposed feature extraction model and failure prognosis model. The second part that contains the next 10 trajectories is used as the verification set to estimate the training results, and the deep forest structure will be preserved when the classification accuracy is greater than 90%. The third part including the remaining 20 trajectories is used as the test set for simulating the real-time condition monitoring processes.

The training, verification, and test sets are composed of 26 columns describing the characteristics of the engine units. The first five columns correspond to the basic information of engine units, such as the engine number, degradation time steps, and constant operational settings, while the remaining 21 columns provide the sensor measurements. Table 1 shows the detailed description of 21 sensor variables, and Table 2 presents part of the condition monitoring data of an engine case. One can see that from the second column of Table 2, the sensor measurements (total temperature at fan inlet) do not change with the increase of operating cycle, and this means that the measurements cannot really reflect the degradation of the engine. In other words, before training the failure prognosis model, it is necessary to extract the representative features reflecting the engine degradation from the measurements of 21 sensors. 

### 3.2. Accuracy of Failure Prognosis Model

To extract the representative degradation features from the 21-sensor data, in the deep auto-encoder modeling, the feature dimensions are set to 4, 8, 12, and 16, respectively. Table 3 reports the prognostic accuracies of different feature dimensions in the cross-validation set. It is observed that when the feature dimension is set to 12, the highest prognostic accuracy can be achieved. In other words, 12 is regarded as a satisfactory feature dimension.

**Figure 6:**
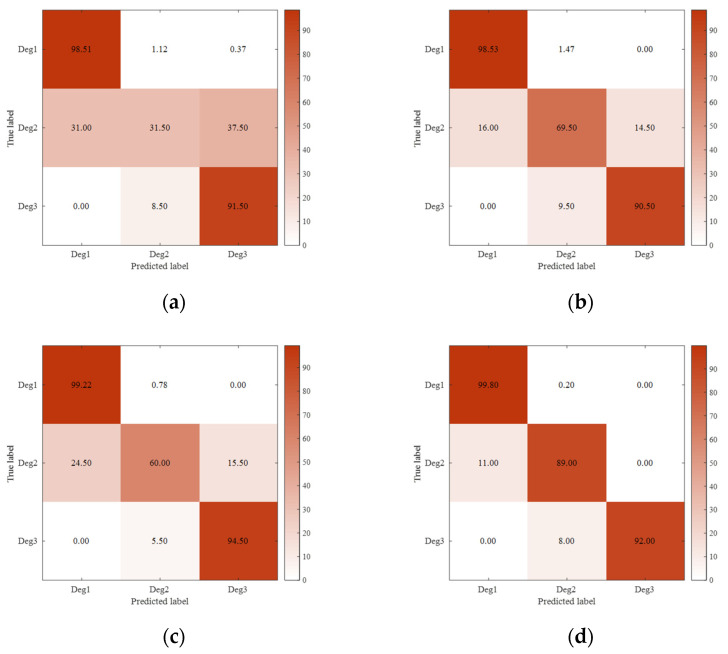
Confusion matrices (%) of different prognostic models on the test engines: (**a**) LSTM network; (**b**) Bi-LSTM network; (**c**) deep forest; and (**d**) deep auto-encoder + deep forest.

Then, the 12 dimensional degradation features extracted from the deep auto-encoder are fed into the deep forest for training. During the training process, if it is found that the prognostic accuracy within 10 running cycles is not improved, the training will stop. Figure 6 presents the average confusion matrices of different prognostic models on the test engines with three respective classes: Deg1 (RUL>20), Deg2 (10<RUL≤20), and Deg3 (RUL≤10). From this figure, no matter which kind of prognostic model (“LSTM network” [24], “Bi-LSTM network” [34], “deep forest” [35], or “deep auto-encoder + deep forest”) is used, the prognostic accuracy for Deg1 is the highest. This can be explained as Deg1 refers to the low degradation, and accordingly there are more training data. Regarding Deg2, the use of the integrated model of a deep auto-encoder and deep forest allows improving the prognostic accuracy. This is mainly because the deep auto-encoder can extract the representative features of engine degradation, while the deep forest can handle the uneven classification sample data. As for Deg3, each model can obtain a prognostic accuracy of more than 90%. Actually, the high prognostic accuracy at this stage is very important for improving the flight safety of the aircraft. In summary, the prognostic model of this paper outperforms the LSTM network, Bi-LSTM network, and single deep forest model.

**Remark** **1.** *It should be noted that in practice, the time windows* $w_{1}$ *and* $w_{2}$ *can be determined according to the requirements of operational planners. In this paper, the values of* $w_{1}$ *and* $w_{2}$ *are assigned to 10 and 20, respectively, to be consistent with the case in [24]. If the proposed failure prognosis method is applied to other cases, these values can still work.*

### 3.3. Performance of the Dynamic Predictive Maintenance Strategy

With the available failure prognostic probabilities, the inventory and maintenance decisions can be made according to the decision rules presented in Section 2.4. Table 4 lists the decision results of some test engines at different inspection moments, given $C_{ins}=0.1$, $C_{os}=10$, $C_{p}=100$, and $C_{c}=500$ [24]. The first column represents the running cycle of the engine, while the second column provides the real RUL according to the “RUL_FD001.txt” text file from the C-MAPSS dataset. The next three columns represent the failure probabilities belonging to different degradation states, while the last three columns represent the order, stock, and maintenance states, respectively.

**Remark** **2.** *It should be noted that these cost parameters, *$C_{ins}$*, *$C_{os}$*, *$C_{p}$*, and*$C_{c}$*, are closely related to the performance of the method. Considering that the failure prognosis-based maintenance strategy presented in [24] has been verified to be flexible under different cost structures, this paper will not repeat the verification. To facilitate the comparative analysis, these parameter values for every case in this paper are set to be the same as those in [24]*.

Considering the engine ID91, one can see that before t=100, the failure probabilities belonging to different degradation states are 96.64%, 3.19%, and 0.17%, which means that the engine is in a low degradation state during operation. Correspondingly, the proposed strategy does not require any maintenance or spare part management activities, and the order, stock, and maintenance Boolean variables are set to be zeros. When t=110, according to Equation (10), it is suggested to order a spare part, and the available spare part will be delivered at t=130. At t=130, due to the high failure probability of Deg3, the optimal decision is to maintain the engine. Regarding the engine ID92, nothing needs to be done before t=310. When t=320, it is suggested to order a spare part, and the available spare part will arrive after two decision periods. However, the engine is required to be maintained at t=330. In this case, one must pay the out-of-stock cost. For the engine ID93, it can be found that there are decision results similar to those of engine 91, and this can be explained by the fact that their life lengths are very close.

**Figure 7:**
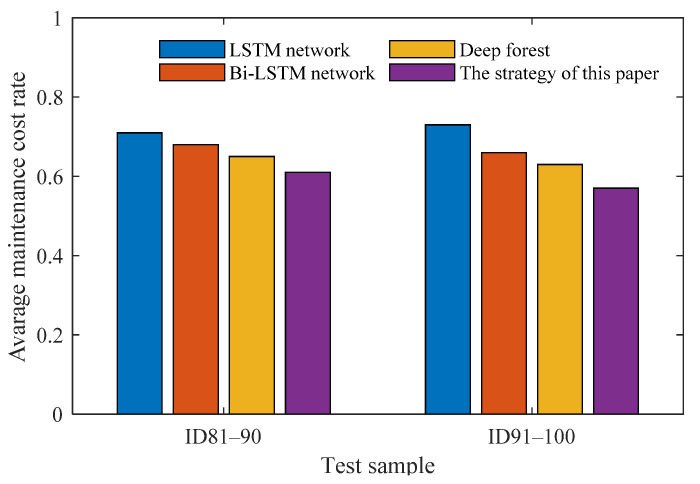
Average maintenance cost rates using different failure prognosis methods for 20 test engines.

Next, the average maintenance cost rates will be used to evaluate the performances of maintenance strategies using different failure prognosis methods, as shown in Figure 7. In this figure, the 20 test engines (ID from 81 to 100) are equally divided into two groups. It is observed that, regardless of the group, the use of deep forest can reduce the average maintenance cost rate of the engines. Further, the inclusion of the deep auto-encoder allows improving the accuracy of decision-making. Accordingly, the strategy of this paper (i.e., “deep auto-encoder + deep forest”-based predictive maintenance) can obtain the lowest maintenance cost rates. 

## 4. Conclusions

In this paper, a deep auto-encoder and deep forest-assisted failure prognosis method was proposed for dynamic predictive maintenance scheduling (DPMS). The representative features reflecting system degradation were extracted from raw sensor data by using a deep auto-encoder, while the inclusion of the deep auto-encoder allowed the nonlinear relationship between the representative features and discrete degradation states to be learned. The integration of these two models led to an effective failure prognosis model. With the prognostic failure probabilities, the maintenance and inventory activities are scheduled through quickly evaluating the costs of different decisions. The verification results using NASA’s open datasets of aircraft engines reveal the feasibility of the proposed DPMS method. According to the prognostic accuracies and average maintenance cost rates, the proposed method is better than several state-of-the-art methods. Future work will focus on the investigation of predictive maintenance strategy for more sophisticated systems with multiple components.

## Figures and Tables

**Table 1:** Description of 21 sensor variables [33].

ID	Symbol	Description	Units
1	T2	Total temperature at fan inlet	ºR
2	T24	Total temperature at LPC outlet	ºR
3	T30	Total temperature at HPC outlet	ºR
4	T50	Total temperature at LPT outlet	ºR
5	P2	Pressure at fan inlet	psia
6	P15	Total pressure in bypass-duct	psia
7	P30	Total pressure at HPC outlet	psia
8	Nf	Physical fan speed	rpm
9	Nc	Physical core speed	rpm
10	epr	Engine pressure ratio (P50/P2)	--
11	Ps30	Static pressure at HPC outlet	psia
12	phi	Ratio of fuel flow to Ps30	pps/psi
13	NRf	Corrected fan speed	rpm
14	NRc	Corrected core speed	rpm
15	BPR	Bypass ratio	--
16	farB	Burner fuel–air ratio	--
17	htBleed	Bleed enthalpy	--
18	Nf_dmd	Demanded fan speed	rpm
19	PCNfR_dmd	Demanded corrected fan speed	rpm
20	W31	HPT coolant bleed	lbm/s
21	W32	LPT coolant bleed	lbm/s

**Table 2:** Sample run-to-failure data from an engine case.

Operating Cycle	Sensor #1 (ºR)	Sensor #2 (ºR)	Sensor #3 (ºR)	⋯	Sensor #21 (lbm·s^−1^)
1	518.67	641.82	1589.70	⋯	23.42
2	518.67	642.15	1591.82	⋯	23.42
3	518.67	642.35	1587.99	⋯	23.34
⋮	⋮	⋮	⋮	⋮	⋮
192	518.67	643.54	1601.41	⋯	22.96

**Table 3:** Prognostic accuracies of different feature dimensions in cross-validation set.

Feature Dimension	Prognostic Accuracy (%)
4	97.23
8	97.73
12	98.13
16	97.55

**Table 4:** Decision results of some test engines at different inspection moments.

Running Cycle	Real RUL	Deg1 (%)	Deg2 (%)	Deg3 (%)	Order	Stock	Maintenance
$\mathrm{Engine}\;\mathrm{ID}91\;T_{f}=135)$							
90	45	96.64	3.19	0.17	0	0	0
100	35	97.01	2.85	0.14	0	0	0
110	25	76.51	22.47	1.01	1	0	0
120	15	37.31	58.54	4.15	1	0	0
130	5	2.29	15.10	82.61	1	1	1
$\mathrm{Engine}\;\mathrm{ID}92\;(T_{f}=341)$							
300	41	94.75	5.02	0.23	0	0	0
310	31	91.33	8.28	0.39	0	0	0
320	21	26.81	65.99	7.20	1	0	0
330	11	6.57	39.58	53.85	1	0	1
$\mathrm{Engine}\;\mathrm{ID}93\;(T_{f}=155)$							
110	45	99.95	0.05	0.00	0	0	0
120	35	99.90	0.09	0.01	0	0	0
130	25	72.29	26.24	1.47	1	0	0
140	15	42.48	53.87	3.65	1	0	0
150	5	1.72	11.24	87.04	1	1	1

## Data Availability

Not applicable.
